# Do Quiet Areas Afford Greater Health-Related Quality of Life than Noisy Areas?

**DOI:** 10.3390/ijerph10041284

**Published:** 2013-03-27

**Authors:** Daniel Shepherd, David Welch, Kim N. Dirks, David McBride

**Affiliations:** 1 School of Public Health, Auckland University of Technology, Auckland 1142, New Zealand; 2 School of Population Health, The University of Auckland, Auckland 1142, New Zealand; E-Mails: d.welch@auckland.ac.nz (D.W.); k.dirks@auckland.ac.nz (K.N.D.); 3 Department of Preventative and Social Medicine, University of Otago, Dunedin 9054, New Zealand; E-Mail: david.mcbride@otago.ac.nz

**Keywords:** quiet, noise, quality of life

## Abstract

People typically choose to live in quiet areas in order to safeguard their health and wellbeing. However, the benefits of living in quiet areas are relatively understudied compared to the burdens associated with living in noisy areas. Additionally, research is increasingly focusing on the relationship between the human response to noise and measures of health and wellbeing, complementing traditional dose-response approaches, and further elucidating the impact of noise and health by incorporating human factors as mediators and moderators. To further explore the benefits of living in quiet areas, we compared the results of health-related quality of life (HRQOL) questionnaire datasets collected from households in localities differentiated by their soundscapes and population density: noisy city, quiet city, quiet rural, and noisy rural. The dose-response relationships between noise annoyance and HRQOL measures indicated an inverse relationship between the two. Additionally, quiet areas were found to have higher mean HRQOL domain scores than noisy areas. This research further supports the protection of quiet locales and ongoing noise abatement in noisy areas.

## 1. Introduction

The protection of living environments is for the public good and a legitimate aim of a democratic society. For example, the European Convention on Human Rights Article 8 states that: “*Everyone has the right to respect for his private and family life, his home and his correspondence*”, and further, that both an individual’s health and wellbeing should be considered in the face of negative environmental factors [1]. Scientific research into the harmful effects of noise exposure is ongoing [2,3,4], and adopting the World Health Organisation’s (WHO) definition of noise, Article 8 can be interpreted as a right of individuals to be protected from noise unnecessarily intruding into their home environments. However, the reality of modern living is that, in many cities and towns, the opportunities to live in homes unencumbered by noise are limited. The European Union indicates that up to 30% of Europeans may be exposed to unsafe levels of noise [5], and that, as a conservative estimate, at least one million healthy life years are lost annually due to traffic noise in Europe alone [4].

Given that noise can adversely impact on health [3,4], and the development of viable models incorporating causal mechanisms representing logical relationships between noise and health [6], it is appropriate that legislative bodies develop policies designed to protect individuals from unnecessary and harmful exposure to noise. Sound is mostly treated as a “waste product”, occupying the first of two approaches to environmental acoustics described by Truax and Barrett: the energy-based *environmental noise management approach* [7]. The other approach, the *subjective listener-centrered* model, encapsulates the concept of the soundscape [8], or the acoustic environment as perceived and understood by people [9]. The EU’s noise directive (2002/49/EC) combines both approaches, being established not only to protect people from harmful noise, but also to identify and guard areas considered quiet. It should be noted that the term “quiet”, is not synonymous with silence; its standard usage implies an absence or masking of industrial noise, and/or the presence of natural sounds such as water flow, birdsong, or wind. Furthermore, the absence of human-generated sounds may be quantified in terms of percentage-time inaudible [10], useful when considering greenbelt, life-style, or rural areas, in which sound pressure measures (e.g., dB(A)) provide inadequate representations of soundscapes [11].

In relation to noise mitigation, it appears that, for the most part, what is judged an acceptable level of noise exposure is largely a societal decision, and not a scientific or legislative one. That is, decisions are made by individuals as to how best distribute resources relative to their needs, by choosing where they live. Consequently, individuals choosing to live in quiet areas can become aggravated when industrialization or other developments threaten their local soundscapes. Unfortunately, however, individuals more susceptible to noise impacts may not always have the financial resources to be able to live in quieter areas, possibly to the detriment of their health and wellbeing. Thus, in many respects, soundscapes can be considered tradable commodities, where tranquility and quiet have value, and noise a cost [12]. For example, research suggests that, all else being equal, a house in the vicinity of a major road can be expected to be worth 8–10% less than a comparable house situated away from the road, with a “noise discount” of around half-a-percent per decibel difference [13], or 1% per dB(A) where noise exceeds 55 dB(A) [14]. Whereas the last centuries have recorded a general social trend towards urbanisation, some are now noting that whilst an urban drift persists, albeit at a reducing rate, a countertrend of ruralisation is also emerging [15]. Such a trend is likely explained by the intrinsic qualities of rural areas, reliably described as “peace and quiet”, and “space and greenness” [15], and some argue that suburban drift can likewise be framed as an effort to move closer to nature [16]. To aid in the protection of quiet areas, and to facilitate access thereof, legislation such as the European Union’s noise directive (2002/49/EC) seeks to preserve quiet areas. Such legislation is guided by an increasing body of evidence indicating the health benefits of quieter areas, and the health costs of noisier areas.

In reading the literature to date, it is apparent that most research focuses directly on the negative impacts of sound (*i.e.*, noise), with relatively fewer studies considering, or directly focusing on, the positive impacts of sound [7]. This “negative” bias to environmental sound is likewise reflected in legislation. For example, in New Zealand, public health and the protection thereof is central to the Resource Management Act (1991; amended 2005) wherein Section 16 describes a “Duty to avoid unreasonable noise”, and Sections 322 to 324 and 326 to 328 of the Act empower local authorities to issue an abatement notice containing the prescribed particulars to an occupier of land from where "unreasonable noise" is emanating [17]. Furthermore, New Zealand’s Health Act (1956) empowers local authorities to abate any nuisance or condition that can be considered either injurious to health or offensive [18]. Noise nuisance is defined by the New Zealand’s Health Act (1956) as “…where any noise or vibration occurs in or is emitted from any building, premises, or land to a degree that is likely to be injurious to health.”, and nuisance more generally as “…a repetitive activity which causes damage to the plaintiff’s land or his enjoyment of it.” [19]. No reference to, and the specific protection of, environmental sounds that promote health and wellbeing can be found in current New Zealand legislation, though more generally, Section 7 (Part C) of the Resource Management Act (1991) directs local authorities to regard the maintenance and enhancement of amenity when managing resources [17].

In considering the legislative approaches embedded in New Zealand policy, it is noted that two common themes emerge from legal approaches to nuisance, namely degraded amenity (*i.e.*, “enjoyment”) or insult to health (*i.e.*, “injurious”). However, these two themes are not mutually exclusive. The WHO defines health as “*a state of complete physical, mental and social well-being and not merely the absence of disease or infirmity*”, where physical, mental and social well-being are themselves dependent upon restorative environments, that is, environments high in amenity. However, the complex interplay between health and amenity is rarely acknowledged in noise-related legislation. Furthermore, the operationalisation of health in the noise context is neither standardised nor straightforward, especially if measurements embody the biomedical approaches emphasis of morbidity and mortality. Normative approaches conceptualise health as optimal functioning relative to sociocultural factors, and relying largely upon self-referential assessments, stipulate health as a precondition of wellbeing, or *health-related quality of life* [20]. Such an approach can, to a greater degree, better document the relationship between sound, amenity, and health by framing specific domains of health-related quality of life (HRQOL) as measuring the influence of an individual’s health status on their global wellbeing. In relation to noise, the WHO (2009) Noise Guidelines (Europe) supports the use of HRQOL measures, stating that “The effects of noise are strongest for those outcomes that, like annoyance, can be classified under “quality of life rather than illness” (p. 92) [3]. The WHO reports that noise-induced annoyance and sleep disturbance can, when chronic, compromise positive wellbeing and HRQOL [2,3,4]. Brown [7] emphasises the positive contribution of soundscapes to quality of life, and identifies significant gaps in research examining the potential restorative value of soundscapes, and their impact upon quality of life and wellbeing.

While investigations into noise and health generally concur that while noise can have a negative impact on populations, the quantification of this impact is made difficult by the multivariate nature of the relationship. Whereas simple bivariate relationships between noise level and human response are no longer considered valid approaches to the protection of the health of the public [21,22], the multiplicity of physical, social, and psychological variables have dictated that other approaches need to be explored. Dratva *et al.* [23] suggest that noise annoyance measures may be superior to noise level when mitigating the harmful impacts of noise. Conversely, low levels (or an absence) of annoyance may be indicative of soundscapes worthy of preserving, especially if they have restorative potential. This exploratory study generates dose (annoyance)-response (HRQOL) functions by utilising data from four New Zealand localities, specifically selected for their differing soundscapes, to further investigate the impacts of sound on health and wellbeing. The study’s main objective can be articulated as the comparison of quiet and noise areas along dimensions of HRQOL.

## 2. Methods

### 2.1. Participants

Data from 823 respondents, taken from four areas, were subjected to analysis. The demographic profile of the sample is displayed in Table 1, accompanied by response rates.

### 2.2. Study Areas

We pooled data from three cross-sectional studies which identified contrasting soundscapes to explore the relationship between noise annoyance and health-related quality of life in adults. The first cross-sectional study explored aircraft noise and contained data collected in July 2009 from residences around the Auckland International Airport [24]. The second study consisted of data collected in July 2010 from two rural samples in the lower half of New Zealand’s North Island, focusing on wind farm noise [25]. The third study reported data also collected in July 2010, but in New Zealand’s largest city, Auckland [26]. The second and third studies utilised a non-equivalent comparison group posttest-only study design, involving the use of “quiet” areas as control groups which were compared to “noisy” areas. For these two studies, strict socioeconomic matching was undertaken using the New Zealand Deprivation Index 2006 [27]. From these three studies, a large dataset (*n* = 823) was formed. The reader is directed to these three papers [24,25,26] for further detail pertaining to sample characteristics and noise levels, and a brief description of the areas now follows:

1. *Quiet Rural* (*n* = 158): residences were in a semi-rural (*i.e.*, greenbelt) area located ten kilometres from New Zealand’s capital city, Wellington, with high social deprivation characteristics, selected for its rural nature and geographic and socioeconomic matching to the Noisy Rural area. Houses were at least eight kilometres from identifiable noise generators.
2. *Noisy Rural* (*n* = 39): residences were located in the Makara Valley, a region located eight kilometres west of Wellington. This area hosts sixty-six 125-metre-high wind turbines, and residences selected for inclusion were within two kilometres of a wind turbine. Wind turbines can be considered noise generators as they are reliably judged as annoying [28] and lacking tranquility [29].
3. *Noisy City* (*n* = 373): residences were located within 50 metres of one of three major motorways as determined by satellite images, or below the flight path of Auckland International Airport’s main runway. These areas were middle-to-high social deprivation areas, with median estimated street noise levels of approximately 76 dB(A) LDN (day and night sound level) near the motorways [26] and between 60 and 65 dB(A) LDN near the airport [30].
4. *Quiet City* (*n* = 253) residences in two areas within Auckland City, with houses at least two kilometres from any motorway, other main roads, or other major sources of environmental noise (e.g., industry). As with the noisy city area, houses in this area could be considered suburban, and were socioeconomically matched to the Noisy City areas. Median estimated street noise levels were approximately 55 dB(A) LDN [26].

### 2.3. Instruments

Questions probing health-related quality of life (26 items), neighbourhood issues (14 items), annoyance to noise and air quality (seven items), demographic information (eight items), and noise sensitivity (one item) were presented in that order. The airport survey did not contain any items probing neighbourhood issues or annoyance to air quality, and differed slightly in terms of demographic questions (see Table 1). Health-related quality of life was assessed using the WHO’s short-form quality of life instrument, the WHOQOL-BREF [31]. This instrument presents two general items on self-rated health and quality of life, and 24 items representing four HRQOL domains: physical health (seven items), psychological wellbeing (six items), social relationships (three items), and environmental factors (eight items). Each WHOQOL item is rated using a five-point scale, with the higher-domain scores indicating more positive evaluations of HRQOL domain scores. It has been proposed that the WHOQOL-BREF is well suited for use in public health research [32], is well validated [31,33], and has been shown to have sound psychometric properties in noise research [26].

Neighbourhood issues were assessed using the neighbourhood problem scale, and played the role of a distracter, designed to mask the intent of the study. Annoyance items asked how annoyed (1 = “Not annoyed at all” to 5 = “Extremely annoyed”) respondents were to air pollution from traffic, residential chimneys, industry or “other (please specify)”, and to noise from traffic, neighbours, or “other (please specify)”. Participants were asked to rate their levels of sensitivity or resistance to noise using a three-point category scale (Not noise sensitive/Moderately noise sensitive/Very noise sensitive), or for the airport sample, the 35 item Noise Sensitivity Questionnaire (NOISEQ) scale was used to estimate a global noise sensitivity score [34].

**Table 1:** Self-reported personal characteristics of the study sample presented by area. Inferential tests, including chi-square (χ^2^) and ANOVA (*F*), were undertaken to test differences across area.

Variables		Rural (Quiet)	Rural (Noise)	City (Noise)	City (Quiet)
		n * (%)	n * (%)	n * (%)	n * (%)
**Response (rate)**		158 (32)	39 (34)	373 (32)	253 (49)
**Sex (χ^2^(3) = 3.488, *p* = 0.322)**					
	Male	63 (41)	16 (41)	117 (31.4)	105 (41.5)
	Female	91 (58)	23 (59)	236 (59.6)	140 (55.3)
**Age group, years (*F*(3,809) = 1.982, *p* = 0.115)**					
	18–20	2 (1.2)	1 (2.6)	10 (2.6)	4 (1.6)
	21–30	1 (0.5)	1 (2.6)	35 (9.4)	14 (5.5)
	31–40	22 (13.9)	5 (12.8)	64 (17.2)	67 (26.5)
	41–50	53 (33.5)	10 (25.6)	68 (18.2)	55 (21.7)
	51–60	44 (27.8)	11 (28.2)	73 (19.6)	40 (15.8)
	61–70	27 (17.1)	7 (17.9)	48 (12.9)	42 (16.6)
	≥71	9 (5.6)	3 (7.7)	50 (13.4)	21 (8.3)
**Education (** **χ^2^(3) = 12.27, *p* = 0.056)**					
	High School	55 (34.8)	11 (28.2)	171 (45.8)	83 (32.8)
	Polytechnic	48 (30.3)	11 (28.2)	95 (25.6)	73 (28.9)
	University	54 (34.2)	17 (43.6)	88 (23.6)	84 (33.2)
**Employment status (** **χ^2^(3) = 29.141, *p* = 0.111)**					
	Full time	83 (52.5)	21 (53.8)	170 (45.6)	126 (49.8)
	Part time	3 (1.8)	0 (0)	47 (12.6)	45 (17.8)
	Unpaid work/Study	3 (1.8)	1 (2.6)	43 (11.5)	24 (9.5)
	Unemployed	27 (17.1)	6 (15.3)	26 (7.0)	12 (4.7)
	Retired	40 (25.3)	10 (25.6)	71 (19.0)	38 (15)
**Noise sensitivity (** **χ^2^(6) = 2.401, *p* = 0.879)**					
	None	60 (37.9)	13 (33.3)	98 (26.3)	94 (37.2)
	Moderate	76 (48.1)	21 (55.3)	211 (56.6)	122 (48.2)
	Severe	20 (12.7)	5 (12.8)	41 (10.9)	25 (9.9)
**Current illness^† ^(** **χ^2 ^(3) = 3.79, *p* = 0.285)**					
	Yes	50 (31.6)	10 (27)	97 (36.2)^ †^	74 (29.2)
	No	104 (65.8)	27 (69.2)	155 (57.8)^ †^	170 (67.2)
**Years of residence (*F*(3,781) = 0.503, *p* = 0.680)**					
	Mean	11.1 (9.9)	12.3 (11.1)	12.6 (12.1)	11.5 (12.5)

* Totals may differ due to missing data. **^†^** Current illness frequencies not available from the airport sample.

### 2.4. Procedure

Each eligible household had two surveys deposited in their letterboxes, along with pre-paid, return-addressed envelopes. A cover sheet explained who was conducting the survey and for what purpose, and invited potential participants to take part in research investigating their place of living and wellbeing. The title of the surveys, “*2010 Wellbeing and Neighbourhood Survey*” was designed to disguise the true intent of the study in order to minimise self-selection biases. Respondents over 18 years of age were invited to participate, completed the surveys independently in their own time and in their own homes, and were offered no incentives for their return.

### 2.5. Analysis

Statistical analyses were performed using the Statistical Package for Social Sciences SPSS version 19, with levels of significance set to α = 0.05. Following a missing-data analysis and the reverse-coding of negatively-worded items, the four WHOQOL-BREF domains were calculated. For composite scales only, internal consistency was verified using Cronbach’s alpha, and item-total correlations were inspected to assess dimensionality. Dose-response relationships were constructed using annoyance as the dose and the WHOQOL-BREF measures as the response variables. Differences between the four areas in terms of HRQOL were assessed using a multivariate analysis of variance (MANOVA), and two-way factorial analyses of variance (ANOVA) were performed to refine the analysis. In keeping with analysis of Dratva *et al.* [23], we dichotomised the sample into “not at all annoyed” (with an annoyance score of “1” for both transport and neighbourhood noise) and “very annoyed” (with an annoyance score of “5” for either transport noise, neighbourhood noise, or both), to further investigate the impact on noise annoyance on HRQOL using an analysis of covariance (ANCOVA) model. As these authors suggest that years of residency impacts on HRQOL scores, this was included in the model as a covariate.

A simple MANOVA was conducted with locality (rural *versus* city) and noise exposure (quiet *versus* noisy) as between-groups factors, and the four WHOQOL-BREF domains (physical, psychological, social, and environmental) constituting the dependent variables. To further explore the impact of locality and noise on HRQOL, a battery of 2 × 2 ANOVA analyses were performed, testing main and simple effects between locality (rural *versus* city) and noise grouping (noisy *versus* quiet) in terms of the WHOQOL-BREF domain scores.

## 3. Results

### 3.1. Analysis of Annoyance Ratings

**Figure 1:**
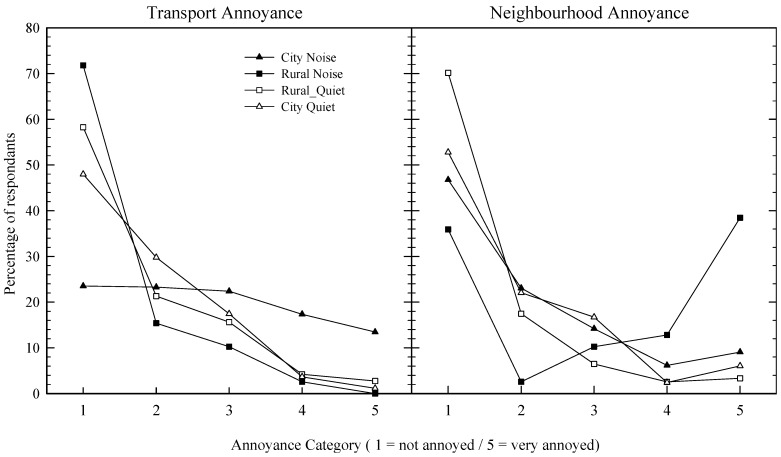
Percentage of respondents indicating annoyance to transportation (left plot) or neighbourhood (right plot) noise, for four different areas (see legend).

Figure 1 shows the five annoyance categories (*x*-axis), and the corresponding proportion of respondents (*y*-axis) for each of the four areas. The left-most plot represents transportation noise, which, for the airport sample, specifically relates to aviation noise, and for the remaining three areas road traffic noise. The right-most plot relates to neighbourhood noise, which can be considered all noise sources other than transport-related noise (e.g., music, barking dogs, industry, and wind turbines). In general, rural areas have lower rates of annoyance towards transportation noise, while severe annoyance to aircraft or road traffic (*i.e.*, noisy city area) is approximately 15% of the exposed sample. In terms of neighbourhood noise, the noisy rural area contains proportionally more annoyed individuals.

**Figure 2:**
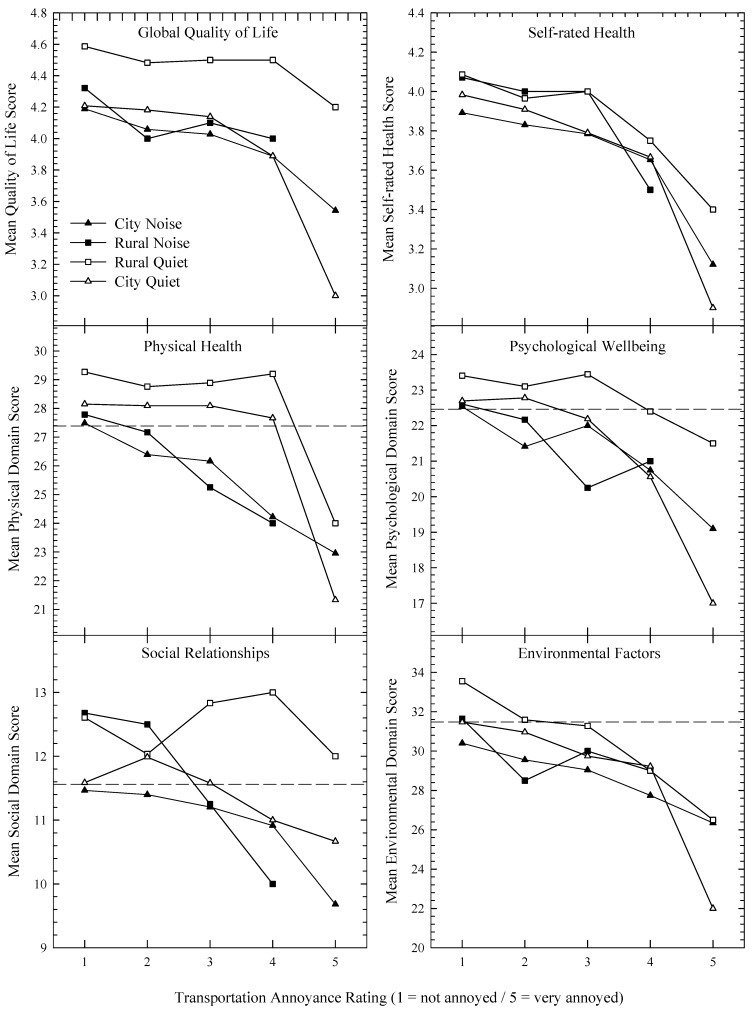
WHOQOL-BREF measures as a function of annoyance to transport noise for the four areas. Note too that the rural noisy area functions plot only four points, as only one individual rated their annoyance as “5”. The dashed-horizontal lines contained within the plots of the four WHOQOL-BREF domains represent national means calculated from a normative sample.

**Figure 3:**
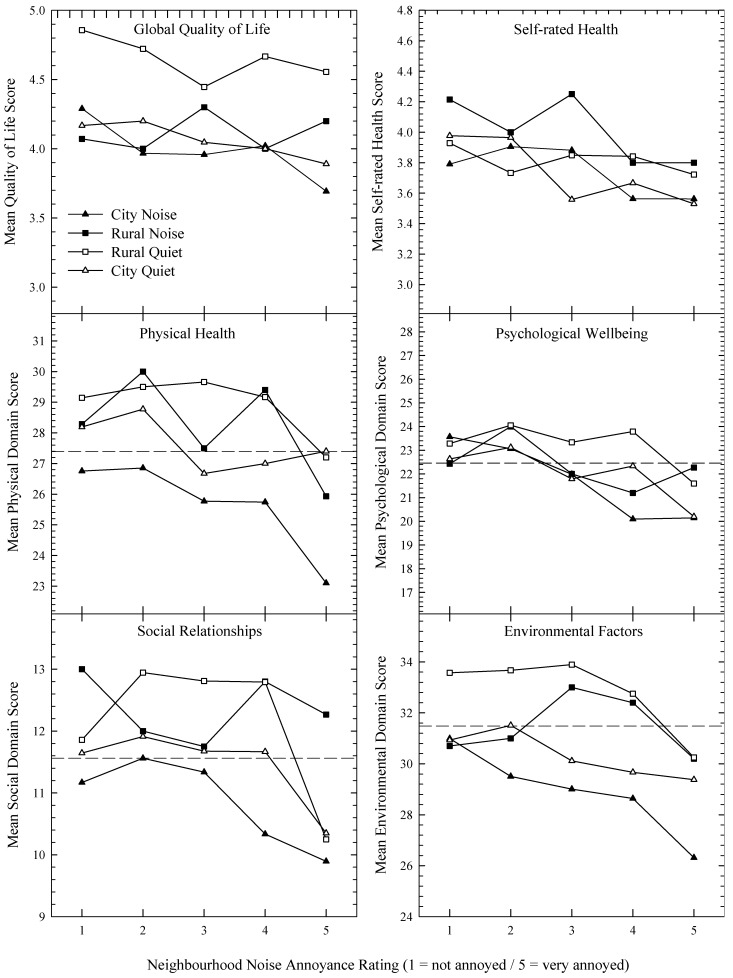
As for Figure 2, but for neighbourhood noise annoyance and the availability of a fifth data point from the rural quiet group.

**Figure 4:**
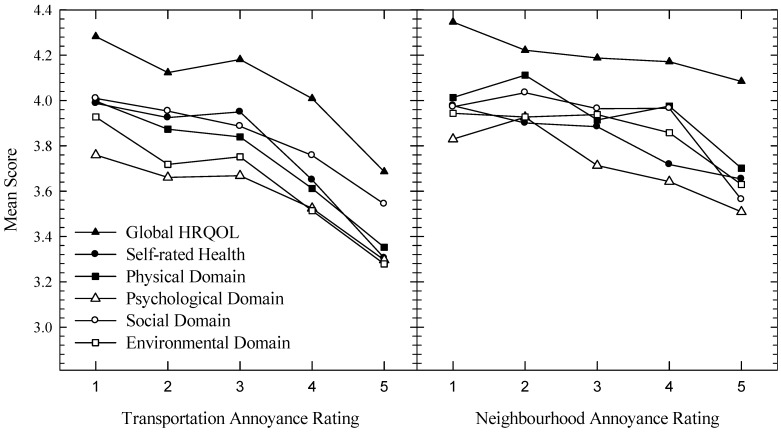
Mean WHOQOL scores plotted as a function of noise annoyance, without regard to area. The left plot is for transportation noise annoyance, the right plot for neighbourhood noise annoyance.

Figure 2, Figure 3 present dose-response curves showing the impact of noise annoyance on HRQOL for the four areas. For Figure 2, all six plots show a noticeable decrease in HRQOL measures as annoyance increased, while for Figure 3 the relationships are not so easy to discern. The dashed horizontal line evident in plots containing the WHOQOL domains represents the average of a New Zealand normative sample [33]. It is evident that as annoyance to transport or neighbours increases, the distance between the plotted data and the normative means increases. Figure 4 pools the data and shows HRQOL scores as a function of transport (left plot) or neighbourhood (right plot) noise without reference to the four areas.

In keeping with the Dratva *et al.*, (2010) analysis, the sample was dichotomized into very annoyed and not at all annoyed. Also, the years of residency was included in the model as a covariate, however, it was found to be non-significant in all cases (*p* > 0.05). The subsequent ANCOVAs showed significant differences between the most annoyed (n = 103) and not annoyed (n = 232) for all domains: physical (*F*(1, 332) = 41.799, *p* < 0.001); psychological (*F*(1, 332) = 36.02, *p* < 0.001); Social (*F*(1, 332) = 14.984, *p* < 0.001), and; Environmental (*F*(1, 332) = 64.83, *p* < 0.001). Pertinently, the most annoyed group had consistently lower mean domain scores than the not annoyed group.

### 3.2. Analysis of Area

**Figure 5:**
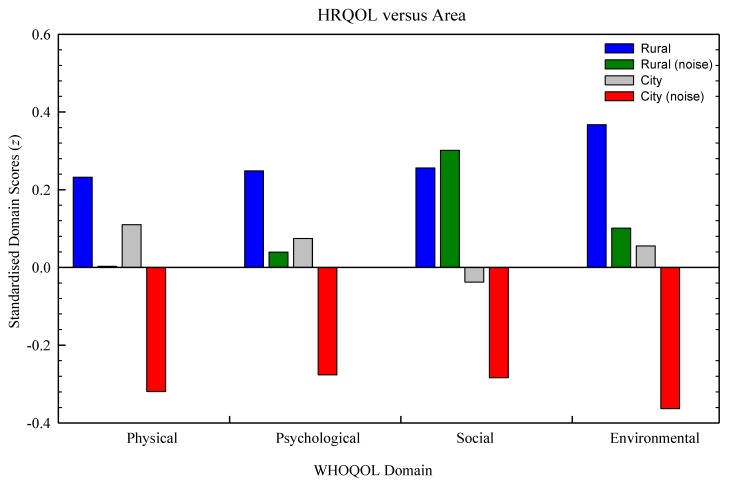
Mean standardised HRQOL scores plotted for the four WHOQOL-BREF domains and four areas differing in sound character.

**Figure 6:**
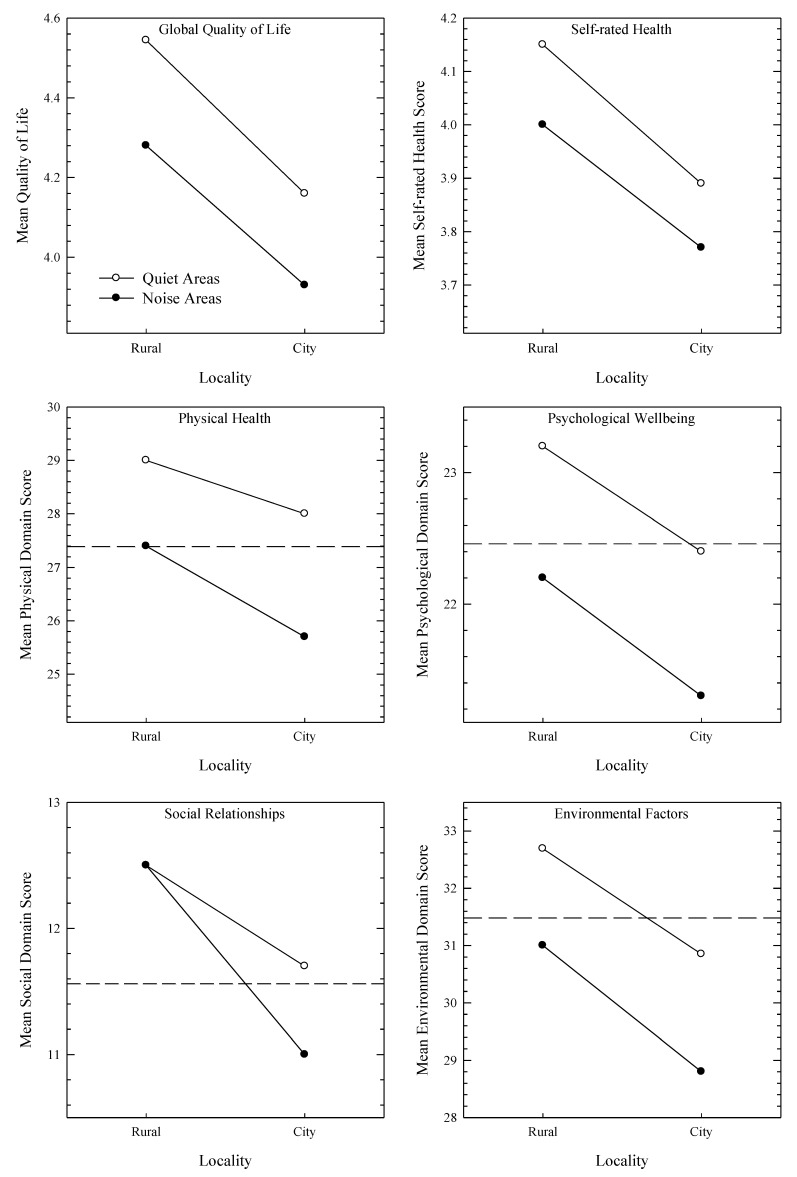
Health-related quality of life (WHOQOL) scores as a function of locality (rural *versus* city) and quiet (open symbols) or noisy (closed symbols) classification. The dashed-horizontal lines contained within the plots of the four WHOQOL-BREF domains represent national means calculated from a New Zealand normative sample.

Figure 5 shows WHOQOL-BREF domain scores (standardised) for each of the four sample areas. Evident is the relatively higher scores of the rural quiet area, and the lower scores of the city noisy area. The rural noise area has lower scores compared to the quiet area in all but the social domain (rural and city quiet) and the environmental domain (city quiet only). Findings from MANOVA analysis with locality (rural *versus* city) and noise exposure (quiet *versus* noisy) as between-groups factors, and the four WHOQOL-BREF domains (physical, psychological, social, and environmental) constituting the dependent variables showed significant findings. There was a significant multivariate effect of the grouped dependent variables in relation to locality (Wilks Lambda = 0.958, *F*(6,806) = 5.948, *p* < 0.001), indicating that HRQOL is related to living environment, and noise (Wilks Lambda = 0.97, *F*(6,806) = 4.124, *p* < 0.001). This suggests that HRQOL is related to noise exposure. However, there was no significant interaction between locality and noise (Wilks Lambda = 0.997, *F*(6,806) = 0.369, *p* = 0.099). To further explore the impact of locality and noise on HRQOL, a battery of 2 × 2 ANOVAs were performed, testing main and simple effects as presented in Table 2. Of remark is the general pattern of significant main effects, with the non-significance between noise areas in relation to social domain scores being the exception, and nonsignificant interaction effects. The trends manifested in Table 2 can be scrutinised graphically in Figure 6, where WHOQOL scores are plotted as a function of locality (rural *versus* city) and quiet or noisy areas.

**Table 2:** *F*-values for tests of main and simple effects between locality (rural *versus* city) and noise grouping (noisy *versus* quiet) in terms of WHOQOL-BREF domain scores.

	Locality	Noise	Locality × Noise
Physical	*F*(1, 818) = 7.905	*F*(1, 818) = 15.659	*F*(1, 818) = 0.275
	*p* = 0.005	*p* < 0.001	*p* = 0.600
Psychological	*F*(1, 818) = 6.677	*F*(1, 818) = 8.708	*F*(1, 818) = 0.105
	*p* = 0.010	*p* = 0.003	*p* = 0.746
Social	*F*(1, 814) = 20.332	*F*(1, 814) = 1.178	*F*(1, 814) = 1.237
	*p* < 0.001	*p* = 0.278	*p* = 0.266
Environmental	*F*(1, 817) = 19.165	*F*(1, 817) = 15.17	*F*(1, 817) = 0.073
	*p* < 0.001	*p* < 0.001	*p* = 0.760

## 4. Discussion

This study examines the relationship between health-related quality of life (HRQOL) and the acoustic environment, with results replicating previous findings of Dratva *et al.* [23] that noise-induced annoyance is both prevalent in areas characterised by unnatural soundscapes and negatively related to HRQOL. We also explored differences across noisy and quiet areas, and uncovered evidence of a positive impact of quiet on HRQOL.

### 4.1. Prevalence of Annoyance

As expected, annoyance to transportation noise was found to depend largely upon exposure, with both rural areas and the quiet city area associated with lower annoyance levels when compared to the noisy city group, a finding reported elsewhere [23,35]. Of those reporting no annoyance to transportation noise, the vast proportion were in the two rural areas, while fifteen percent of the noisy city sample indicated that they were very annoyed with transportation noise, a figure that concords to European estimates indicating that between 10% and 35% of city dwellers are severely annoyed or very annoyed by road traffic noise [23,35,36,37]. The differences in transportation-induced noise annoyance between rural and city areas found in the current study and by others [23] contribute to the general perceptions of green (quiet, spacious and restorative) and city (noise, crime, crowding, and stressful) areas [16], suggesting that “quiet” is a core characteristic of green areas.

For neighbourhood noise, we found a similar pattern between the quiet rural area and the two city areas (quiet and noisy); by proportion, the rural area has less annoyance to noise than the two city areas. However, this relationship is not found when the industrialized noisy rural area is referenced; there are a greater proportion of very annoyed respondents than in the rural quiet area or the two city areas. Data indicating lower levels of noise annoyance in rural areas reflect general descriptors of such localities as bastions of “peace and quiet” [12], containing sounds that are generally preferred, such as moving water and other natural sounds [7]. However, when these preferred sounds are masked by other, more mechanical sounds, then an individual’s “sense of place” may be reduced and annoyance will likely result. Brambilla and Maffei [11] demonstrate that the more a sound is congruent with the context in which it is heard, the more it is deemed acceptable, with congruence being largely dependent upon individual expectations. Thus, for green areas, the introduction of non-natural sounds can be expected to induce annoyance by violating the expectations of residents and/or masking pleasant sounds.

An alternative approach formulates the notion of a “listening radius” in quiet rural areas (Rick James, personal communication), especially at night, allowing people to feel connected to distant parts of their community. For example, the sound of a distant neighbouring farmer’s children playing outside at night may reduce feelings of isolation. As rural residents hear the activities of the community from miles around, they are connected to places and activities inaccessible to other senses. When industrial plants are installed and the night-time soundscape is dominated by mechanical noise, the listening radius shrinks substantially, and fainter sounds and those from greater distances are lost. This makes the sense of place not much different from suburban or even urban living. As the listening radius collapses, individuals come to exist in a smaller-and-smaller bubble, isolated within their homes, and relying upon televisions or radiograms to mask the unwanted sounds dominating outside. Thus, when industrialization takes place, such as with the positioning of wind turbines in our rural noise area, a rural community may lose that part of the soundscape containing the small auditory cues that make rural homes seem like part of a natural environment and integrated community. The listening radius approach echoes the concepts of “behaviour settings” described by Barker [38], and is interesting inasmuch as it endows an intrinsic value on the soundscape that cannot be logically related to objective noise units, such as the decibel or Hertz, at least beyond the concept of audibility [7,11].

### 4.2. Relationship between Annoyance and HRQOL

Our dose-response functions (transportation-induced noise annoyance as a function of HRQOL) presented in Figure 2 demonstrate the same trends as those reported by Dratva *et al.* [23], and, as such, can be considered a replication of their data. Firstly, as annoyance increases, HRQOL generally decreases. Secondly, while some data appear to be sufficiently described by linear functions, others are better approximated by nonlinear or piecewise linear relationships. Third, some HRQOL domains in some areas manifest breakpoints, potentially providing noise tolerance thresholds. Additionally, our analysis adds to that of Dratva *et al.* [23] in that while they reported significant differences in annoyance across localities (e.g., village/city/rural), these differences were not intensively explored when describing the relationship between annoyance and HRQOL, even though they included locality in their model. Evident in our findings (Figure 2) are the generally higher levels of HRQOL in the quiet rural sample compared to the rest. This may be explained by a number of factors, including less frequent (but equally intense) annoyance responses to transportation noise, a general reluctance to report health difficulties in rural populations [39], or that living in a rural area has a buffering effect against the negative impacts of noise. Thus, the retention of the locality variable in the analysis may yield a richer insight into the relationship between HRQOL and noise annoyance, which as seen in Figure 4 (plotting mean WHOQOL scores as a function of annoyance), may be lost when locale data are aggregated.

For neighbourhood noise (Figure 3), the functions are not as clearly defined as those obtained with the transportation noise data. This could possibly be due to other characteristics of the noise, such as the number of noise events [40], which may be quite variable. This is in contrast with transportation noise which typically occurs in predictable quantities. Thus some noise sources (e.g., parties, barking dogs, lawn mowers) may be highly annoying when heard, but as the incidences are limited, they may not substantially affect HRQOL. Note, however, that when the data are aggregated across area (see rightmost plot, Figure 4), the functions become sufficiently linear, and so the variability evident in Figure 3 may reflect either genuine acoustic and population characteristics across locality, or an attenuation of the influence of extreme scores as the sample size increases. Interestingly, Figure 4 suggests that the mean HRQOL domain scores are equally impacted by noise annoyance, at least when the slope of the functions between the third and final points are visually assessed.

### 4.3. Quiet versus Noisy Areas

Our analyses of areas differing in sound profile present evidence of a positive impact of soundscape upon HRQOL. For all but the social domain, the mean WHOQOL scores were significantly greater in areas classified as quiet. Thus, in noisy areas, the character of the soundscape could potentially be exerting a negative effect on health by inducing annoyance, disrupting sleep, or by masking sounds that would otherwise be restorative. The negative impacts of noise upon health and wellbeing are well recognised [4,41] though the characterization of the positive impacts of wanted sound and the scientific analysis of such sounds is a more recent mainstream interest [42]. There is evidence suggesting that quiet areas make a positive contribution to public health, especially for those individuals regularly exposed to noise [16]. It has been argued that natural environments can facilitate restorative physiological processes by inducing positive emotions [43] or by directing attention from fatigue and reducing mental exhaustion [44], or perhaps a combination of the two [45]. Fredrickson *et al.* [46] propose that positive emotions reverse the cardiovascular insults of negative emotions, and a number of other studies provide empirical support for the restorative influence of natural environments, including enhanced recovery from physiological stress, improvement of health and well-being, and decreased negative affects [43,47,48]. Unfortunately, our own data cannot determine the relative contributions of noise exposure, on the one hand, and impeded restoration through the masking of positive sounds, on the other, upon HRQOL. Further studies and methodological approaches need to be developed in this area.

Furthermore, there was no interaction effect between rural and city localities, suggesting that differences in HRQOL are not simply due to the costs-and-benefits of living in one locality or the other, but are related to differences in the sound environments. This interpretation is supported by the inclusion of a noisy rural area in which a previously quiet rural area had become industrialised. A difficulty when comparing quiet areas to noisy areas is that, invariably, factors other than soundscapes change need to be accounted for, and the comparison of two rural areas differing mainly in their soundscapes affords greater confidence in attributing a causal relationship. Additionally, comparisons are available to New Zealand WHOQOL normative data, available for the four HRQOL domain, as indicated in Figure 6. Here, the two noisy areas (rural and city) are equivalent to, or below the normative data, in all four domains except in the social domain in which the rural noise area is higher.

### 4.4. Limitations and Future Research

Our results suggesting that quiet areas afford greater HRQOL than noisy areas must be interpreted within the limitations of the data. Firstly, we did not undertake physical sound surveys of the localities, though surveys undertaken by others were consulted when selecting localities. This may not necessary be a limitation, as others (e.g., [23,49]) argue, pervasively in our view, that noise annoyance is the correct measure with which to investigate noise-induced stress, as it better accounts for human factors. Indeed, Zwicker [24] questions the “enthronement” of the dB(A) scale in environmental noise management, demonstrating that in many contexts, dB(A) measures are of no intrinsic use, and can produce misleading assessments. In warning against the exclusive use of physical sound measures in noise control situations, one of Zwicker’s [21] statements is worth repeating here (p. 67):

*“It is* *definitely not the simple dB(A) measuring equipment which is annoyed by the noise, but individuals and their hearing organs that have to endure the noise whether they like it or not!”*

A second limitation is that our results, when comparing across locales, do not disentangle the impacts of noise through sleep disturbance or general annoyance, and critically, the interaction between the two. In this regard, future research would benefit from including measures of sleep quality and the adoption of more rigid theoretical models (e.g., [32]) to guide data collection and analyses. For example, the notion that perceptions of amenity may moderate annoyance has been raised elsewhere (e.g., [25]), and is an important variable when rural and green areas are studied. Thirdly, our cross-sectional design, and analysis of a modestly-sized dataset, does not provide sufficient criteria in which to judge causality. Thus, like most studies of this type, we cannot conclude definitively that noise annoyance is degrading HRQOL, and that quiet is the guardian of such. However, we can reference other similar but more analytically amenable datasets, such as that of Dratva *et al.* [26], whom with over 5,000 respondents reported comparable results, even after controlling for scores of variables including pre-existing health status. A final consideration is that, due to inherent differences between the demographic structures of rural and city areas, comparisons between rural areas, and between city areas will be more reliable than comparison between rural and city, due to matching characteristics.

## 5. Conclusions

We present exploratory data indicating that, relative to noisier areas, quiet areas facilitate restoration, or impede insult, to health as reflected by HRQOL measures. Modern living is challenging, and managing stress is essential to health and wellbeing. Research is increasingly showing that disagreeable soundscapes can induce annoyance or sleep disruption, whilst positively evaluated soundscapes can aid restoration. Our results add to the small number of studies offering quantitative evidence of the benefits afforded by quiet areas. Given the value that individuals’ place on green and quiet areas [50], even when located within city limits [51], and the possible restorative features of these areas, legislation such as the European Noise Directive can be considered progressive and justified. Data such as we present further justifies the establishment of legislation protecting quiet areas, and that limiting access to such areas, especially in compact cities, may not be in the best interests of public health.

## References

[1] Case of Hatton and Others *v.* The United Kingdom. http://airportnoiselaw.org/cases/hatton-1.html.

[2] Berglund B., Lindvall T., Schwela D.H. (1999). Guidelines for Community Noise.

[3] (2009). Night Noise Guidelines for Europe.

[4] (2011). Burden of Disease from Environmental Noise.

[5] Unsafe Levels of Noise in Europe: European Union (2000). https://osha.europa.eu/en/publications/reports/6905723.

[6] Andringa T., Lanser J. Sound Annoyance as Loss of Options for Viability Self-26 Regulation. Proceedings of the 10th International Congress on Noise as a Public Health Problem 27 (ICBEN).

[7] Brown A.L. (2012). A Review of progress in soundscapes and an approach to soundscape planning. Int. J. Acoust. Vib..

[8] Schafer R.M. (1977). The Tuning of the World.

[9] Axelsson Ö. (2011). Progress in soundscape research requires a common agenda. J. Acoust. Soc. Am..

[10] Miller N.P. (2008). US National Parks and management of park soundscapes: A review. Appl. Acoust..

[11] Brambilla G., Maffei L. (2006). Responses to noise in urban parks and in rural quiet areas. Acta Acust. United Ac..

[12] van Dam F., Heins S., Elbersen B.S. (2002). Lay discourses of the rural and stated and revealed preferences for rural living: Some evidence of the existence of a rural idyll in the Netherlands. J. Rural Stud..

[13] Nelson J.P. (1982). Highway noise and property values: A survey of recent evidence. J. Transport Econ. Pol..

[14] Milieu Ltd., Risk and Policy Analysis Ltd. Final Report on Task 2: Inventory of Potential Measures for a Better Control of Environmental Noise. www.nho.cz/knihy/end_task_2_final_report.pdf.

[15] van Dam F., Haartsen T., Groote P., Huigen P.P.P. (2000). Revealed and Stated Preferences for Rural Living. Evidence from the Netherlands. Claiming Rural Identities: Dynamics, Contexts, Policies.

[16] Van den Berg G.P., Pedersen E., Bouma J., Bakker R. (2008). Project WINDFARMperception. Visual and Acoustic Impact of Wind Turbine Farms on Residents. FP6-2005-Science-and-Society-20. Specific Support Action Project No. 044628. Final Report.

[17] Resource Management Act 1991. www.legislation.govt.nz/act/public/1991/0069/latest/DLM230265.html.

[18] (1956). Powers and Duties of Local Authorities. New Zealand’s Health Act (1956).

[19] Panckhurst G. (2000). Langdon v Bailey, AP3-00: Timaru Registry.

[20] Salomon J.A., Mathers C.D., Chatterji S., Sadana R., Ustun T.B., Murray C.J.L., Murray C.J.L., Evans D.B. (2003). Quantifying Individual Levels of Health: Definitions, Concepts, and Measurement Issues. Health Systems Performance Assessment: Debates, Methods and Empiricism.

[21] Zwicker E., Fastl H. (1999). Psychoacoustics: Facts and Models.

[22] Fidell S. (2003). The Schultz curve 25 years later: A research perspective. J. Acoust. Soc. Am..

[23] Dratva J., Zemp E., Dietrich D.F., Bridevaux P.O., Rochat T., Schindler C., Gerbase M.W. (2010). Impact of road traffic noise annoyance on health-related quality of life: Results from a population-based study. Q. Life Res..

[24] Shepherd D., Welch D., Dirks K.N., Mathews R. (2010). Exploring the relationship between noise sensitivity, annoyance and health-related quality of life in a sample of adults exposed to environmental noise. Int. J. Environ. Res. Public Health.

[25] Shepherd D., McBride D., Welch D., Dirks K.N., Hill E.M. (2011). Evaluating the impact of wind turbine noise on health-related quality of life. Noise Health.

[26] Welch D., Shepherd D., Dirks K.N., McBride D. (2013). Road traffic noise and health-related quality of life: A cross-sectional study. Noise Health.

[27] Ministry of Health New Zealand Deprivation Scores, 2006. www.moh.govt.nz/moh.nsf/indexmh/dhb-maps-and-background-information-atlas-of-socioeconomic-deprivation-in-nz-nzdep2006.

[28] Janssen S.A., Vos H., Eisser A.R., Pedersen E. (2011). A comparison between exposure-response relationships for wind turbine annoyance and annoyance due to other sources. J. Acoust. Soc. Am..

[29] Pheasant R.J., Fisher M.N., Watts G.R., Whitaker D.J., Horoshenkov K.V. (2010). The importance of auditory-visual interaction in the construction of “tranquil space”. J. Environ. Psychol..

[30] (2009). Auckland International Airport Limited. Annual Aircraft Noise Contours. www.aucklandairport.co.nz/Corporate/Social-Responsibility/Sustainability-policy/Environmental-management/Noise/Annual-Aircraft-Noise-Contours.aspx.

[31] Skevington S.M., Lotfy M., O’Connell K.A. (2004). The World Health Organization’s WHOQOL-BREFquality of life assessment: Psychometric properties and results of the international field trial—A report from the WHOQOL group. Q. Life Res..

[32] Lercher P. (1996). Environmental noise and health: An integrated research perspective. Environ. Int..

[33] Krägeloh C.U., Kersten P., Billington R., Hsu P., Shepherd D., Landon J., Feng X. (2012). Validation of the WHOQOL-BREF quality of life questionnaire for general use in New Zealand: Confirmatory factor analysis and Rasch analysis. Qual. Life Res..

[34] Schutte M., Marks A., Wenning E., Griefahn B. (2007). The development of the noise sensitivity questionnaire. Noise Health.

[35] Paunovic K., Jakovljevic B., Belojevic G. (2009). Predictors of noise annoyance in noisy and quiet urban streets. Sci. Total Environ..

[36] Skinner C.J., Ling M.K., Grimwood C.J., Raw G.J. (2002). United Kingdom Results. The 1999/2000 National Survey of Attitudes to Environmental Noise.

[37] Fyhri A., Aasvang G.M. (2010). Noise, sleep and poor health: Modelling the relationship between road traffic noise and cardiovascular problems. Sci. Total Environ..

[38] Barker R.G. (1968). Ecological Psychology: Concepts and Methods for Studying the Environment of Human Behavior.

[39] Kroneman M., Verheij R., Tacken M., van der Zee J. (2010). Urban-rural health differences: Primary care data and self reported data render different results. Health Place.

[40] Basner M., Müller U., Griefahn B. (2010). Practical guidance for risk assessment of traffic noise effects on sleep. Appl. Acoust..

[41] Ising H., Kruppa B. (2004). Health effects caused by noise: Evidence in the literature from the past 25 years. Noise Health.

[42] Truax B., Barrett G.W. (2011). Soundscape in a context of acoustic and landscape ecology. Landsc. Ecol..

[43] Ulrich R.S., Simons R.F., Losito B.D., Fiorito E., Miles M.A., Zelson M. (1991). Stress recovery during exposure to natural and urban environments. J. Environ. Psychol..

[44] Kaplan R., Kaplan S. (1989). The Experience of Nature.

[45] Chang C., Hammitt W.E., Chen P., Machnik L., Su W. (2008). Psychophysiological responses and restorative values of natural environments in Taiwan. Landsc. Urban Plan..

[46] Fredrickson B.L., Mancuso R.A., Branigan C., Tugade M.M. (2000). The undoing effect of positive emotions. Motiv. Emot..

[47] Grinde B., Patil G.G. (2009). Biophilia: Does visual contact with nature impact on health and well-being?. Int. J. Environ. Res. Public Health.

[48] Parsons R., Tassinary L.G., Ulrich R.S., Hebl M.R., Grossman-Alexander M. (1998). The view from the road: Implications for stress recovery and immunization. J. Environ. Psychol..

[49] Cox T. (2010). Sound effects and the city. New Sci..

[50] Wiersinga W. (2008). Compensation as a Strategy for Improving Environmental Quality in Compact Cities.

[51] Bonaiuto M., Aiello A., Perugini M., Bonnes M., Ercolani A.P. (1999). Multidimensional perception of residential environment quality and neighborhood attachment in the urban environment. J. Environ. Psychol..

